# Impact of a school-based water, sanitation, and hygiene intervention on school absence, diarrhea, respiratory infection, and soil-transmitted helminths: results from the WASH HELPS cluster-randomized trial

**DOI:** 10.7189/jogh.09.020402

**Published:** 2019-12

**Authors:** Anna N Chard, Joshua V Garn, Howard H Chang, Thomas Clasen, Matthew C Freeman

**Affiliations:** 1 Department of Environmental Health, Rollins School of Public Health, Emory University, Atlanta, Georgia, USA; 2 School of Community Health Sciences, University of Nevada Reno, Reno, Nevada, USA; 3 Department of Biostatistics, Rollins School of Public Health, Emory University, Atlanta, Georgia, USA

## Abstract

**Background:**

Water, sanitation, and hygiene (WASH) in schools is promoted by development agencies as a modality to improve school attendance by reducing illness. Despite biological plausibility, the few rigorous studies that have assessed the effect of WASH in schools (WinS) interventions on pupil health and school attendance have reported mixed impacts. We evaluated the impact of the Laos Basic Education, Water, Sanitation and Hygiene Programme – a comprehensive WinS project implemented by UNICEF Lao People’s Democratic Republic (Lao PDR) in 492 primary schools nationwide between 2013 and 2017 – on pupil education and health.

**Methods:**

From 2014-2017, we conducted a cluster-randomized trial among 100 randomly selected primary schools lacking functional WASH facilities in Saravane Province, Lao PDR. Schools were randomly assigned to either the intervention (n = 50) or comparison (n = 50) arm. Intervention schools received a school water supply, sanitation facilities, handwashing facilities, drinking water filters, and behavior change education and promotion. Comparison schools received the intervention after research activities ended. At unannounced visits every six to eight weeks, enumerators recorded pupils’ roll-call absence, enrollment, attrition, progression to the next grade, and reported illness (diarrhea, respiratory infection, conjunctivitis), and conducted structured observations to measure intervention fidelity and adherence. Stool samples were collected annually prior to de-worming and analyzed for soil-transmitted helminth (STH) infection. In addition to our primary intention-to-treat analysis, we conducted secondary analyses to quantify the role of intervention fidelity and adherence on project impacts.

**Results:**

We found no impact of the WinS intervention on any primary (pupil absence) or secondary (enrollment, dropout, grade progression, diarrhea, respiratory infection, conjunctivitis, STH infection) impacts. Even among schools with the highest levels of fidelity and adherence, impact of the intervention on absence and health was minimal.

**Conclusions:**

While WinS may create an important enabling environment, WinS interventions alone and as currently delivered may not be sufficient to independently impact pupil education and health. Our results are consistent with other recent evaluations of WinS projects showing limited or mixed effects of WinS.

School-aged children in low-income settings are at substantial risk for water, sanitation, and hygiene (WASH)-related infections such as pathogens causing diarrheal diseases, soil-transmitted helminths (STH), and trachoma [1-4]. Crowded, unsanitary conditions may facilitate the spread of pathogens, and increase pupils’ risk for disease [5]. Improved access to WASH facilities combined with sufficient behavior change may not only prevent the spread of pathogens within the school domain but also lead to beneficial WASH habits at home and throughout the life course [5-8]. The limited data available indicate that only 69% of schools worldwide have access to sanitation facilities, while only 66% have access to water [9]. WASH in schools (WinS) targets and indicators have been included in the Sustainable Development Goals [10].

Despite the biological plausibility of WinS interventions to reduce illness and subsequently school absence, evidence of impact has been mixed. Some WinS efficacy studies, such as those assessing intensive handwashing programs in China and Egypt, reported reductions in absence and absence due to illness. However, with only 6- and 3-month follow up periods, respectively, and with soap being continuously supplied by the intervention or school administration, respectively, the long-term sustainability of handwashing behaviors linked to these impacts is unknown [11,12].

Effectiveness trials of WinS projects have not replicated this success. A matched-control evaluation of a comprehensive WinS program in Mali revealed reductions in pupil-reported diarrhea, symptoms of respiratory infection, and absence due to diarrhea, but higher odds of absence overall among pupils enrolled in beneficiary schools. However, there were imbalances between the beneficiary and comparison groups at baseline, and the study was further limited by inconsistent fidelity to the intervention by implementing partners and participating schools [13]. A randomized controlled trial (RCT) of a WinS program in Kenya reported a 44% reduction in odds of *Ascaris lumbricoides* reinfection, but no overall impact on absence or diarrhea. Program impact differed by intervention arm (as individual and combined WASH interventions were employed) and subsets of the sample population [14-16]. Absence among girls in the hygiene promotion and water treatment arm reduced by 58% [16]. In water-scarce schools that received a comprehensive WASH intervention, including water supply improvements, risk of diarrhea among pupils reduced by 61% [15], while diarrhea among pupils’ siblings under 5 years old reduced by 56% [17]. However, program impact may have been affected by incomplete and inconsistent intervention delivery (fidelity) and uptake and use by the target population (adherence) [15,16]. A WinS intervention in Lao People’s Democratic Republic (Lao PDR), Cambodia, and Indonesia had no impact on STH infection or being underweight, but reported evidence of improvement in dental cavities. Again, this evaluation was potentially limited by incomplete fidelity and adherence to the program, as well as a non-randomized design and contamination from concurrent programming in control schools [18].

Here, we present results from the Water, Sanitation, and Hygiene for Health and Education in Laotian Primary Schools (WASH HELPS) study, a cluster-RCT designed to measure the impact of a comprehensive WinS project – water supply, sanitation, handwashing, and behavior change - in Lao PDR on pupil absence, diarrhea, respiratory infection, and STH infection. Given past challenges in program fidelity and adherence to project outputs and behaviors [19], we also apply two analyses that have previously been used to evaluate the role of intervention fidelity and adherence on WinS project impacts [20,21].

## METHODS

### Study setting and intervention

The Laos Basic Education, Water, Sanitation and Hygiene Programme was implemented by UNICEF in 492 primary schools across thirteen provinces between 2013 and 2017. The WASH HELPS Study, a research component of the intervention, was conducted between September 2014 and May 2017 in Saravane Province, which was selected because it was the only province in which intervention activities had not yet occurred, thus allowing a randomized intervention trial.

The study setting, baseline results, intervention components, intervention outputs and outcomes, and their fidelity and adherence have been described in detail elsewhere [19]. Key outputs and outcomes of the project are listed in **Table 1**. Briefly, the comprehensive WinS project included provision of a school water supply, sanitation facilities, handwashing facilities (individual and group), drinking water filters, and behavior change education and promotion. The project was implemented in two phases; lessons learned from Group 1 schools (n = 52; intervention started in 2014) were applied to improve the project for Group 2 schools (n = 48; intervention started in 2015), leading to different levels of achievement at output and outcome levels between groups, as well as different durations of follow-up [19].

**Table 1:** Intervention outputs and behavioral outcomes and their measurement indicators

Output	Indicator and criteria
Water supply	• Improved* water point on school compound
	- Water point functional in the previous year (director reported)
	• Water tank to supply toilet and handwashing stations
	- Water observed in tank
Toilets	• At least one improved* toilet compartment
	- Toilet is sex separated (by designation)
	- Toilet is unlocked
	- Toilet is clean
	- Toilet has water available inside compartment for flushing
Handwashing facilities	• At least one individual handwashing station available to pupils
	- Water available at individual handwashing station
	- Soap available at individual handwashing station
Promotion of daily group hygiene activities	• Daily group handwashing schedule posted in at least one classroom or near toilet
	• Daily group compound cleaning schedule posted in at least one classroom or near toilet
	• Daily group toilet cleaning schedule posted in at least one classroom or near toilet
Group handwashing	• Group handwashing facility available to pupils
	- Water available at group handwashing facility
	- Soap available at group handwashing facility
Water filters	• At least one drinking water filter available in a classroom for pupil use
	- Water in filter
**Outcome**	**Indicator**
Toilet use	• Percentage of students using toilet for defecation during school hours (pupil-reported)
Handwashing (individual)	• Percentage of students washing hands with soap and water upon exiting toilet (observation)
Daily group handwashing	• School conducted daily group handwashing the day of visit (observation)
Daily group toilet cleaning	• Percentage of students participating in daily group toilet cleaning within the previous five school days (pupil-reported)
Daily group compound cleaning	• Percentage of students participating in daily group compound cleaning within the previous five school days (pupil-reported)

*Defined according to Joint Monitoring Programme (JMP) standards.

### Study design, sampling, and data collection

We conducted a cluster-randomized, controlled trial among 100 primary schools (50 intervention, 50 comparison). Study design, sampling, and data collection methods have been previously published [19].

We used stratified random sampling to help ensure equal representation of control and intervention schools in each district, and that the number of schools selected in each district was proportional to the number of eligible schools in each district. We selected up to 40 pupils from grades 3-5 in each school using systematic stratified sampling, with grade and sex as the stratification variables. Pupils selected at baseline were followed throughout the entire study period; pupils who left the school due to abandonment or transfer were replaced at the beginning of the following academic year, maintaining equal grade and sex ratios when possible. Pupils who progressed from fifth to the sixth grade were replaced with pupils from grade three the following academic year. A total of 3993 pupils were enrolled throughout the study period.

Data were collected over three or two school years (Group 1 and 2 schools, respectively) to measure uptake and sustainability of facilities and behavior change. To account for variabilities across time and season, data were collected throughout the school year, which consists of 33 weeks across two semesters (September-January and February-May), with five to six hours of instruction per day [22]. Trained enumerators visited study schools every six to eight weeks during the school year through March 2017, for a total of 11 (Group 1) or 7 (Group 2) visits per school. All visits were unannounced and during school hours. At each visit, enumerators conducted a roll call of all students enrolled in the school using sex- and grade-specific ledgers; interviewed the school directors; interviewed sampled pupils in grades 3–5; observed conditions and functionality of WinS hardware; and observed individual and group handwashing practices. Each year, stool samples were collected from up to 50 pupils per school prior to distribution of preventative chemotherapy as part of the National School Deworming Programme. Stool samples were tested for *Ascaris lumbricoides*, *Trichuris trichuria*, and hookworm (*Ancyclostoma duodenale* and *Necatur americanus*) using the Kato Katz technique [23].

### Measures

Our primary impact of interest was pupil absence measured by school-wide roll-call at each visit. At the beginning of each data collection visit, enumerators visited each classroom with a roster of all students enrolled in the school, stratified by grade and sex. At each visit, enumerators confirmed with the head teacher whether there were any new students since the last visit or if any students had left the school. New students were added to the roster. Dropout was recorded for students who had dropped out since the last visit. Absences that were followed by a designation of dropout or transfer were removed from roll-call analysis.

Secondary educational impacts included enrollment, dropout, and progression. Enrollment was calculated at each visit by summing the count of pupils on the roll-call roster and subtracting those who had dropped out or transferred. In addition to student-level dropout recorded in the roll-call register, an aggregate school-level count of dropout was reported by the school at the end of each school year. Pupils who transferred to another school were not considered to have dropped out. Progression was school-reported at the end of each academic year as the count of students who passed the national exam and progressed to the next grade level. All secondary educational impacts were stratified by grade and sex.

Secondary health impacts included diarrhea, symptoms of respiratory infection, and conjunctivitis/non-vision related eye illness and were collected through pupil interviews. All health impacts were binary and self-reported with a one week recall period. Pupils were asked if they had had diarrhea using local terminology and were also asked how many times they had defecated each day; a pupil was considered to have had diarrhea if he or she had reported having diarrhea and had defecated three or more times in a 24-hour period [13,15,24]. Pupils were considered to have symptoms of respiratory infection if they reported cough, runny nose, stuffy nose, or sore throat [13]. During the last visit we included negative-control questions about self-reported cuts/scrapes and toothache. These questions served as a measure of respondent bias, as there is no biological plausibility of an association between a WinS program and cuts/scrapes or toothache [25]. Data on STH infection were collected yearly. Any sample testing positive for the hookworms, *A. lumbricoides*, or *T. trichuria* considered positive for STH infection.

Intervention fidelity and adherence for this study has been described previously [19]. To measure fidelity- defined as how the intervention was delivered per the stated design- we created an index score in which one point was given for each of the 20 output criteria fulfilled (**Table 1**). For each visit, the minimum intervention fidelity score was zero and the maximum score was 20. To measure adherence – defined as achievement of behavioral outcomes promoted by the intervention – a similar index score was created. Although there were five behavioral outcomes of interest (**Table 1**), we excluded group compound cleaning from the index given that reported participation in group compound cleaning was nearly universal among both intervention and comparison schools at baseline (97.9%) [19], therefore the adherence score ranged from 0-4. A behavior was considered to be achieved when >75% of pupils reported or were observed to complete the behavior except for group handwashing, which was binary (either the school performed group handwashing or did not).

### Analysis

Data were analyzed using Stata Statistical Software: Release 13 (StataCorp LP, College Station, TX, USA) and SAS version 9.4 (SAS Institute, Cary, NC, USA).

#### Intention to treat analysis

Our primary analysis was an intention-to-treat (ITT) analysis, which was used on all primary and secondary impacts. For binary impacts (roll-call absence, diarrhea, symptoms of respiratory infection, conjunctivitis/non-vision related eye illness, STH infection, toothache, cuts/scrapes), we estimated relative risk using a “modified Poisson” approach. This is a validated method to produce relative risk ratios for binary data using a multi-level mixed Poisson model with robust error variances [26], and was chosen for this analysis because Stata does not support the use of log-linear binomial regression when using mixed effects generalized linear models. Odds ratios were obtained when the modified Poisson model did not converge for a specific impact (eg, toothache). Random intercepts at the school and pupil levels were included to account for clustering of pupils within schools and for repeated measures of pupils over time, respectively. For count impacts (enrollment, abandonment, progression), we estimated relative risk using Poisson regression models. As these data were aggregated at the school-level, we included a random intercept at the school level only.

All ITT models compared intervention schools to comparison schools as they were randomly allocated to intervention and comparison groups, without regard to project fidelity or adherence. Intervention and comparison schools were balanced on key indicators at baseline [19], therefore intervention schools were included in the analysis once UNICEF documented that full intervention implementation (eg, both hardware and behavior change components) was complete. Since full implementation generally occurred at the same time in each district, comparison schools were also included once implementation occurred in their respective districts.

Models included several design variables, including the district and visit number, and controlled for the following fixed effects, determined *a priori* based on biological plausibility of affecting impacts: pupil sex, pupil grade, school enrollment size, season (rainy or dry). The rice crop calendar (planting, growing, harvesting) was included as a fixed effect in the absence model because rice agriculture is the predominant economic activity in the province and the need to stay home and support the family was the leading cause of pupil-reported absence. Fully adjusted models were used to produce adjusted risk ratios (aRR) for each of the associations of interest. These fixed effects, as well as whether the school was concurrently receiving aid from the World Food Program (WFP) school feeding program, were also assed for effect modification. Covariates were determined to be effect modifiers if an interaction term between the covariate and intervention group was significant in the full model.

Intervention fidelity and adherence are important considerations when evaluating the impact of WASH programs. Assessing these factors along the causal ‘theory of change’ allows us to understand not only *if* but *why* and *how* that intervention succeeded or not in that context (ie, was there theory failure [27]?). Further, assessment of the process can determine if the intervention followed the intervention protocol to activate that theory of change (ie, was there intervention failure?). In contexts where fidelity and adherence to the intervention is imperfect, ITT results may underestimate the causal effect for the potential impact of changes to outputs or outcomes, resulting in null or mixed effects [28,29]. Given the suboptimal fidelity and adherence of the intervention based on our monitoring data [19], we conducted a secondary analysis to quantify the impact of the project as implemented by UNICEF and adhered to by schools and pupils on the primary impact (roll-call absence) and select secondary impacts (diarrhea, respiratory infection, and STH prevalence). We explore two modeling frameworks that have been previously used to evaluate the role of fidelity and adherence to a school WASH intervention on project impacts: As-treated (AT) analysis and Structural Nested Models (SNMs) [20,21]. Each framework operates under different assumptions and differ in robustness and efficiency; as such, comparing estimates lends a more informed picture of project impact [30].

**Figure 1:**
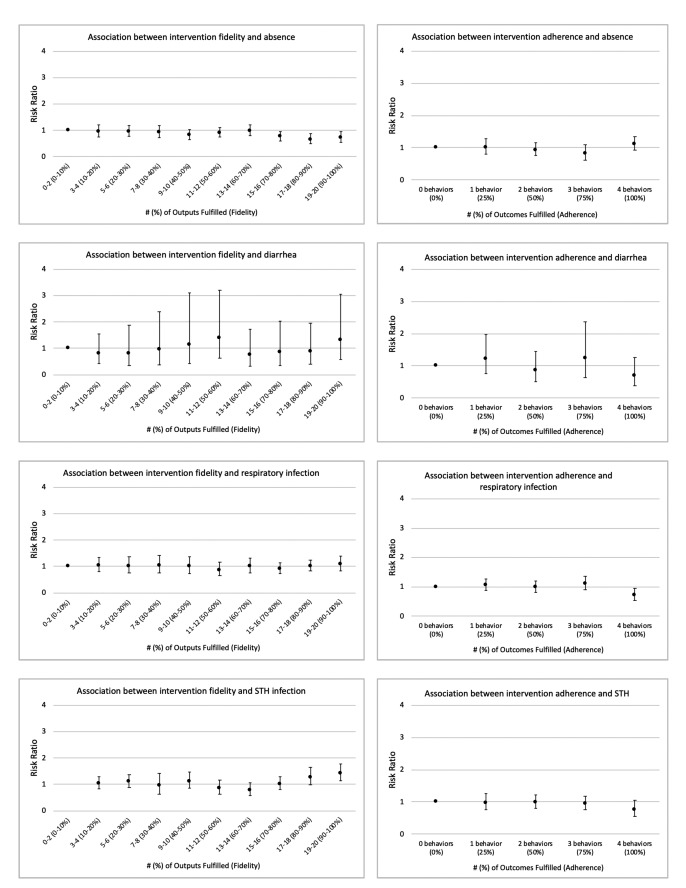
Association between intervention fidelity and adherence continuum and intervention impacts.

We conducted a sensitivity analysis to identify a meaningful threshold of fidelity and adherence. The scale of 20 outputs (fidelity) were categorized with cut-points at each 10th percentile and the scale of four outcomes (adherence) were unadjusted. We observed lower risk of absence among schools with 70%-80% intervention fidelity and higher, but there was no clear evidence of a threshold for any other association (**Figure 1**). We thus selected a threshold of 75%, which is consistent with previous research on fidelity to WinS projects [20]. Only the SNM requires specifying a threshold of fidelity/adherence, however, we also applied the 75% threshold to the AT models for comparability between the two approaches.

#### As-treated analysis

The AT analysis groups subjects according to the treatment *received* and does not consider the treatment *intended* (as is the case with ITT analysis). Advantages to the AT approach are that it is analytically straightforward and easily supports our clustered and longitudinal study design. Disadvantages are that characteristics of schools with good fidelity or students who adhere to behaviors may be fundamentally different from those with poor fidelity/adherence, which can lead to confounding. This confounding may be remedied by controlling for the prognostic factors that led participants to choose to adhere, but only if those prognostic factors are known, which is often not the case [31].

For the AT analysis, we ran two separate models that were structurally identical to the ITT models. However, instead of using intervention status as the primary predictor, as in the ITT analysis, schools were grouped according to intervention fidelity (ie, fulfilling ≥75% of outputs or not) and adherence (ie, fulfilling ≥75% of outcomes or not), respectively. AT models included the same covariates as the ITT models, with *a priori* identified fixed effects and random intercepts at the school and pupil levels. Only data collected after the implementor reported intervention delivery was complete were included. AT models were stratified by effect modifiers identified in the ITT analysis.

#### Structural nested model analysis

Second, we assessed the role of fidelity and adherence using SNMs, an instrumental variable approach. SNMs resolve the potential confounding issue presented by AT models because they do not break the randomization of intervention status [30]. Instead, SNMs create a counterfactual for each study participant in order to compare the risk of an impact among adherers against the risk of the impact had the same individual not adhered [20]. Unlike the ITT and AT models, to control for relevant covariates, a weighted distribution of population data are produced in order to remove the association between population-level confounders and randomization [20,32]. While SNMs are advantageous because they account for unknown or unmeasured confounders, drawbacks are that they are more computationally intensive and rely on strong assumptions. SNM assumptions are described in detail elsewhere [20,32]; briefly, they are as follows: (1) Exclusion restriction – randomization has no direct effect on the outcome; (2) Consistency – observed outcomes are possible under the fidelity/adherence level actually observed; (3) The potential outcomes used to estimate the SNM effects are independent of randomization; (4) No interaction – the model’s causal effect is consistent across randomization groups.

Our code was derived from Garn et al [20] and adapted for a 2-arm trial. Because the SNM methodology we used does not accommodate repeated measures, we averaged time-varying pupil-level data (eg, grade, absence, reported diarrhea, reported symptoms of respiratory infection) and school-level data (output index, behavior index) across the data collection period. As such, binary variables such as absence, reported diarrhea, and reported symptoms of respiratory infection became a continuous variable between zero and one, in which zero indicated never being absent, reporting diarrhea, or reporting symptoms of respiratory infection, whereas one indicated always being absent, reporting diarrhea, or reporting symptoms of respiratory infection. Similar to the ITT and AT models described above, observations were included only after full implementation had been achieved. Models were adjusted using the same covariate variables as we used in the ITT and AT models. As with the AT models, achievement of ≥75% of outputs and ≥75% of outcomes were considered achieving fidelity and adherence, respectively. SNMs were stratified by effect modifiers identified in the ITT analysis [20].

For all analyses, results were considered statistically significant if the *P*-value was <0.05.

### Ethics

The WASH HELPS Study was approved by Emory University’s Institutional Review Board (IRB0076404) and the Lao Ministry of Health’s National Institute of Public Health National Ethics Committee (No. 043 NIOPH/NECHR). Both Institutional Review Boards approved consent *in loco parentis* (in the place of the parent) signed by the school director. Pupils who were selected for the pupil interview and/or stool collection provided informed verbal assent prior to any data collection. All consent/assent procedures occurred after randomization. The intervention was delivered to comparison schools in April 2017, after research activities ended. The study is registered in ClinicalTrials.gov (NCT02342860).

## RESULTS

### Baseline results and intervention fidelity and adherence

**Figure 2:**
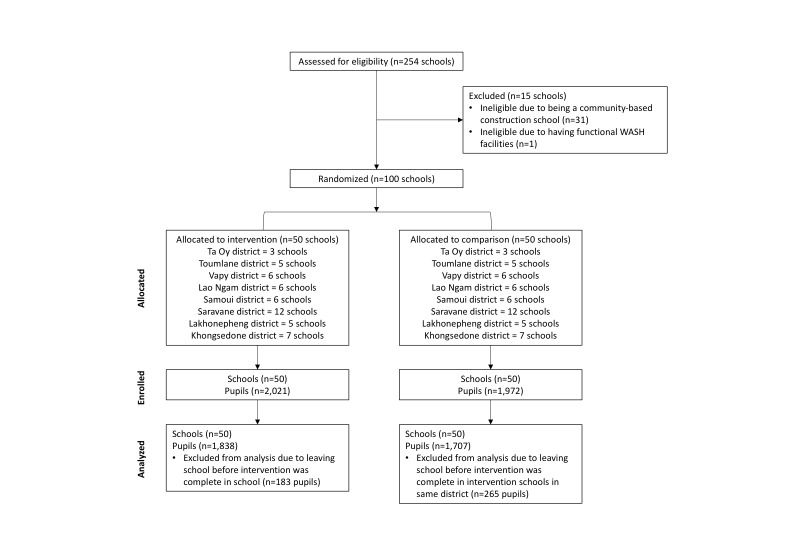
Flow diagram of school and pupil selection.

A total of 100 schools (n = 50 intervention, n = 50 comparison) were randomized, received the intervention, and included in the analysis (**Figure 2**). There were no significant differences in key pupil-level or school-level indicators between intervention and comparison groups at baseline, indicating that the cluster-randomization was successful in creating balanced groups [19]. Following full intervention implementation, intervention fidelity was 30.9% across all schools and visits and intervention adherence was 29.4%. Data on fidelity to specific project outputs and adherence to specific project behaviors across the evaluation period have been previously published [19].

### Intention-to-treat analysis

We found no impact of the intervention on the primary impacts (roll-call absence) or secondary impacts (enrollment, progression, pupil-reported diarrhea, pupil-reported symptoms of respiratory infection, pupil-reported conjunctivitis, STH infection; **Table 2**).

**Table 2:** Association between WinS intervention and health and educational impacts, Saravane Province, Lao People’s Democratic Republic, 2014-2017 (n = 100 schools)

Impact	Comparison*	Intervention*	Adjusted risk ratio	95% confidence interval
Roll-call absence†	6024 (32.2%)	7147 (29.9%)	1.01	0.84, 1.20
Enrollment**‡**	68.2 (49.7)	71.6 (50.0)	1.07	0.84, 1.35
Dropout**‡**	0.8 (2.6)	0.4 (1.0)	0.56	0.25, 1.24
Grade progression**‡**	64.4 (48.6)	67.3 (48.6)	1.07	0.91, 1.25
Diarrhea†,§	1032 (21.1%)	947 (14.7%)	0.80	0.51, 1.26
Symptoms of respiratory infection†**,‖**	1414 (28.9%)	2064 (32.1%)	1.08	0.95, 1.23
Conjunctivitis†,§	41 (0.8%)	48 (0.8%)	0.89	0.53, 1.52
Prevalence of any STH†,¶	1833 (39.8%)	1935 (41.6%)	1.00	0.85, 1.17

*Data are n (%) for impacts at the pupil level (roll-call absence, diarrhea, symptoms of respiratory infection, conjunctivitis, and prevalence of STH) and mean (SD) for impacts at the school-level (enrollment, dropout, grade progression) across study period.

†Risk ratios were calculated using a Poisson model with robust error variances and random intercepts at the school and pupil level. All models adjusted for district, visit number, pupil sex, pupil grade, school enrollment size, and season (rainy or dry). Absence model additionally controlled for and rice crop calendar (planting, growing, harvesting).

**‡**Risk ratios were calculated using a Poisson model with random intercepts at the school level. All models adjusted for district and visit number.

§Pupil-reported in previous week.

**‖**Pupil-reported cough, runny nose, stuffy nose, or sore throat in previous week.

¶Samples testing positive for *Ascaris lumbricoides*, *Trichuris trichuria*, and hookworm (*Ancyclostoma duodenale* and *Necatur americanus*).

There was some evidence of effect modification. Risk of diarrhea was higher in the rainy season compared to the dry season; when stratified by season, there was no significant impact of the intervention on diarrhea in either season (Dry season adjusted risk ratio (aRR) = 0.69, 95% confidence interval (CI) = 0.44, 1.10; Rainy season aRR = 1.14, 95% CI = 0.65, 1.99). Pupil sex, pupil grade, school enrollment size, receiving support from the WFP school feeding program, and the rice crop calendar (absence model only) did not modify the effect of any primary or secondary impacts.

We found no difference in reported prevalence of toothache or cuts/scrapes (the negative control questions) among pupils attending intervention vs comparison schools (toothache adjusted odds ratio (aOR = 0.64, 95% CI = 0.23, 1.84; cuts/scrapes aRR = 1.06, 95% CI = 0.66, 1.72), indicating that any respondent bias that may have been present occurred equally between groups.

### As-treated analysis

AT results are presented in **Table 3**. Intervention fidelity – meeting ≥75% of output indicators associated with water supply, toilets, handwashing facilities, promotion of group hygiene activities, group handwashing facilities, and filtered drinking water– was associated with roll call absence and prevalence of STH. Compared to students attending schools without intervention fidelity, students attending schools with intervention fidelity had a 23% lower risk of absence (aRR = 0.76, 95% CI = 0.64, 0.91) and a 20% higher risk of STH prevalence (aRR = 1.20, 95% CI = 1.01, 1.43). Diarrhea was significantly higher during the rainy season, but when stratified there was no significant difference by fidelity status (Dry season aRR = 0.84, 95% CI = 0.48, 1.49; Rainy season aRR = 1.65, 95% CI = 0.82, 3.33).

**Table 3:** Association between WinS intervention fidelity and adherence and absence, diarrhea, respiratory infection, and soil-transmitted helminth infection (STH), Saravane Province, Lao PDR, 2014-2017 (n = 100 schools)

	As-treated analysis		Structural nested model analysis	
	Adjusted risk ratio*	**95% confidence interval**	**Adjusted risk ratio†**	**95% confidence interval**
**Roll-call absence:**				
Fidelity**‡**	0.76	0.64, 0.91	0.97	0.33, 2.81
Adherence**‡**	0.91	0.79, 1.05	0.96	0.19, 4.97
**Diarrhea:**§				
Fidelity, dry season**‡**	0.84	0.48, 1.49	0.45	0.24, 0.85
Adherence, dry season**‡**	1.00	0.70, 1.44	0.42	0.21, 0.87
Fidelity, rainy season**‡**	1.65	0.82, 3.33	1.03	0.42, 2.51
Adherence, rainy season**‡**	1.41	0.61, 3.26	0.50	0.19, 1.30
**Symptoms of respiratory infection‖:**				
Fidelity**‡**	1.00	0.89, 1.14	1.41	0.93, 2.13
Adherence**‡**	0.97	0.84, 1.11	2.30	0.54, 8.87
**Prevalence of any STH:**¶				
Fidelity**‡**	1.20	1.01, 1.43	1.10	0.57, 2.13
Adherence**‡**	0.93	0.77, 1.12	1.18	0.37, 3.73

STH – soil-transmitted helminth

*Risk ratios calculated using a Poisson model with robust error variances and random intercepts at the school and pupil level. All models adjusted for district, visit number, pupil sex, pupil grade, school enrollment size, season (rainy or dry). Absence models additionally controlled for rice crop calendar (planting, growing, harvesting).

†Risk ratios calculated using a Structural Nested Model with random intercepts at the school level. All models adjusted for district, visit number, pupil sex, pupil grade, school enrollment size.

**‡**Fulfilling ≥75% of intervention outputs was considered fidelity. Fulfilling ≥75% of intervention outcomes was considered adherence.

§Pupil-reported in previous week.

**‖**Pupil-reported cough, runny nose, stuffy nose, or sore throat in previous week.

¶Samples testing positive for *Ascaris lumbricoides*, *Trichuris trichuria*, and hookworm (*Ancyclostoma duodenale* and *Necatur americanus*).

Intervention adherence – meeting outcome indicators associated with toilet use, handwashing with soap after toilet use, daily group handwashing, and daily group toilet cleaning – was not significantly associated with any impacts.

### Structural nested model analysis

Results from the SNMs are presented in **Table 3**. Diarrhea was the only impact associated with fidelity or adherence. When stratified by season, diarrhea was lower in the dry season among students attending schools with intervention fidelity (aRR = 0.45, 95% CI = 0.24, 0.85) and adherence (aRR = 0.42, 95% CI = 0.21, 0.87); there was no significant difference in diarrhea between groups during the rainy season.

## DISCUSSION

In the primary analysis, we found no evidence that the intervention had an effect on absence, school enrollment, dropout, grade progression, pupil-reported diarrhea, pupil-reported symptoms of respiratory infection, pupil-reported conjunctivitis, or prevalence of STH. These results contribute to the growing body of research showing limited or mixed impacts of WinS effectiveness trials on pupil health and education [13-16,18]. Since 2010, access to WASH has been a fundamental human right recognized by the United Nations General Assembly [33]. As such, regardless of its potential education and health impacts, WinS access is an important objective, evidenced by its inclusion in the Sustainable Development Goals [10]. However, if improvements in education and health indicators are to be achieved, results from this and other rigorously evaluated WinS programs suggest that WinS interventions *alone*, and as currently delivered in many contexts, may be insufficient to achieve anticipated education and health impacts.

The theory of change for WinS programs posits that improved WASH access leads to reductions in pathogen exposure at the school level and the habitualization of hygiene behaviors that can be practiced both at school at and home, which in-turn leads to reduced illness and thus reduced school absence [8]. Numerous factors influence school absence, such as household wealth, distance to school, and number of siblings [34]. Lao PDR is a least-developed country, with over 65% of the population working in agriculture [35]. In Saravane Province, where over half of the population lives in poverty [35], the school calendar largely coincides with rice planting and harvesting seasons, and children are often kept home from school to assist in the fields and with other household chores [22]. Indeed, in the current study, the leading pupil-reported cause of school absence was the need to stay home to support the family in economic activities (9.4% of pupils in intervention group and 8.7% of pupils in comparison group across all visits), not illness (5.1% of pupils in intervention group and 5.8% of pupils in comparison group across all visits), which may explain the lack of an impact of the intervention on absence. Thus, the role of school WASH in supporting an enabling environment may be critical, but ultimately not sufficient to reduce absence when other factors like household economic needs or food security is the main driver of truancy from school. Complementary approaches to WinS may be necessary to achieve improvements in absence and other educational impacts. For example, WinS may be successful in combination with school feeding programs [36] or conditional cash transfers [37], both of which have been associated with reduced absence and increased enrollment in other low- and middle-income contexts. Although our results did not reveal a significant interaction between the WFP school feeding program and absence or enrollment, our study was not designed or adequately powered to detect a difference.

Although there are potential mechanisms by which improved WASH may impact illness independently of measurable impacts on absence [13], we found no overall impact of the WinS intervention on pupil illness. These results contrast to previous WinS research that reported overall reductions in diarrhea [13], respiratory infection [13], and absence due to illness [11,12], but are consistent with results from a WinS intervention in Lao PDR, Cambodia, and Indonesia that found no impact of the intervention on STH or underweight [18]. One explanation for the lack of an effect of the WinS intervention on pupil illness is low household WASH access; in this study context, the health benefits linked to improvements in school WASH conditions and behaviors provided by this intervention were likely not sufficient to overcome other potential transmission pathways at home or elsewhere in the community. Environmental improvements in both the domestic and public domains may be required for successful control of infections targeted by environmental improvements, such as diarrhea [38]. As such, WinS alone may not achieve significant health gains without concurrent community and household WASH improvements.

Fidelity and adherence are fundamental antecedents to achieving intervention effects. It is possible that the lack of an effect of the intervention could be due, in part, to sub-optimal or unsustained fidelity and adherence. However, our secondary analyses yielded limited evidence of an effect of the intervention, even at high levels of intervention fidelity and adherence. Additionally, our sensitivity analysis showed no clear trend in impacts across the fidelity/adherence continuum. With two exceptions – the association between fidelity and lower absence (AT analysis) and the association between fidelity and adherence and lower diarrhea during the dry season (SNM analysis) – we did not find that fidelity and adherence led to improved education or health. These results support the above conclusion that factors other than WinS – such as low household WASH access or household economics – may supersede health and education benefits of a WinS intervention in low-income contexts.

However, the AT evidence should be should be interpreted cautiously due to the limited potential for causal inference resulting from breaking the randomization assignment in the AT analysis. The two fidelity and adherence analyses results were inconsistent and sometimes yielded estimates of effect in opposite directions (eg, associations between adherence and diarrhea, respiratory infection, and STH), which is likely due to unaccounted for confounding in the AT analysis. IV analyses are known to yield estimators with high variance, especially when compliance is low [30], which may also partially explain differences between the AT and SNM results. The choice of which method to use depends on numerous factors, including study design, plausibility of meeting analysis assumptions, and available analytical resources; our conflicting estimates highlight the importance of testing the sensitivity of multiple fidelity analysis options [30].

### Strengths and limitations

The design, methods and approach of the WASH HELPS study were robust. Randomized controlled trials offer the greatest potential for causal inference. The longitudinal design allowed us to collect data across three full school years of in Group 1 schools and two full school years of in Group 2 schools, allowing us to capture inter-seasonal and inter-year variations in the outputs, outcomes, and impacts. All data were collected during unannounced school visits so that schools could not prepare for the visit and bias observations. Our primary measure of impact – roll-call absence - is an objective measure of school absence. This impact evaluation was conducted by external researchers, to foster an unbiased assessment of the project impact. Our field team was composed of experienced Laotian enumerators to ensure the tools were designed and delivered with cultural and contextual appropriateness. This robust study design lends strong internal validity, and results may be generalized to the larger, nationwide WinS project. This was an effectiveness trial evaluating an intervention as conducted in a real-world setting. The lessons from this project, taken with other recent WinS trials, reveal heterogeneity of findings that can inform programming across contexts. Lastly, in addition to comparing two methods to analyze the effect of intervention fidelity on WinS impacts, our fidelity analysis also examines adherence to intervention behaviors, which has not been previously included in WinS fidelity analyses.

There are a number of limitations to this evaluation. First, the secondary health impact measures (diarrhea, symptoms of respiratory infection, conjunctivitis) were based on self-report by pupils, which may be subject to bias, and this evaluation was not blinded for either the beneficiaries or data collectors. More objective and robust measures of pupil health, such as molecular methods to detect enteric infection in stool samples, would improve our confidence in the reported impacts, though these measures can be costly, time consuming, and require specialized equipment and laboratory staff. As a way to measure potential reporting bias, we included a negative control question about symptoms of illness unrelated to WASH access (cuts/scrapes and toothache) at the last survey visit. Differences in reported symptoms of these illnesses between intervention and comparison groups would indicate a potential reporting bias, but we found no evidence to suggest that any bias may have existed to a greater degree among either the intervention group or the comparison group. Additionally, schools in the comparison group did not have functional WASH facilities, so it is unlikely that the null results could be explained by a change in behaviors among the comparison group. Second, the intervention was delivered across two different school years, so Group 1 schools had one more year of surveillance than Group 2 schools. Following a single cohort of schools over the same time period would have provided a more accurate measure of WinS hardware and software performance, sustainability, and impact. Third, implementation was delayed in many Group 1 schools. The intervention was fully implemented in Group 1 schools at visit 4, with the exception of Samoui district, in which the intervention was fully implemented at visit 9 [19]. Our analysis excludes visits prior to full intervention implementation, thus power may have been limited by dropping observations under incomplete intervention delivery. Last, we were unable to account for the quality of intervention design or dose of the intervention received, which are important components of fidelity and adherence [39,40].

## CONCLUSIONS

Our findings and those of other rigorous WinS trials suggest that WinS programs – as currently designed and delivered – do not have a population-level benefit on education and health. In this context, the WinS improvements alone were not sufficient to address the other powerful causes of absenteeism, enrollment, and dropout that are not related to – but possibly more influential than – school WASH. We believe this likely holds in many similar settings. Similarly, WinS improvements, though potentially critical for the enabling environment [7], may not be sufficient to overcome disease transmission in areas where community and household WASH coverage is poor. WinS, independent of its stated purpose of improving education and health, is an important objective for dignity, inclusivity, and development. However, if intended impacts are to be achieved, improving intervention fidelity and adherence and including other complementary approaches for WASH may be required. To better understand how to improve intervention fidelity and adherence, evaluations of WinS interventions need to better understand and adapt to contextual drivers of key impacts and outcomes, further develop and test theories of change, and conduct rigorous process evaluations to understand where along the causal pathways interventions are falling short.
